# A Legal View of "Pure and Wholesome Water"

**Published:** 1901-02-02

**Authors:** 


### A LEGAL VIEW OF "PURE AND WHOLESOME WATER."

A case came before the Railway and Canal Commission last week which led to a somewhat interesting judgment—one which serves well to illustrate some of the difficulties which stand in the way of those who would insist on the water companies furnishing the inhabitants of London with a pure and wholesome supply of drinking water. The Vestry of St. Mary, Battersea, complained that the water supplied for domestic use to the inhabitants of Battersea on many occasions was not "effectually filtered," nor "pure and wholesome" within the meaning of the statute. Since the year 1892 forty-five samples of water had been analysed on behalf of the vestry, of which nine had been reported on as being "bad" and four as "inferior." The water reported on as bad contained "infusoria, diatoms, and other living organisms," whose presence rendered the water unwholesome and liable to give rise to various water-borne diseases. Now it would seem to most reasonable people that if it could be proved that these living organisms, many of which are of comparatively large size, can pass through the filters, all sorts of smaller organisms, including any pathogenic microbes which might happen to be present, might also obtain access to the water as supplied for drinking purposes. One cannot but feel then that the evidence given by Sir William Crookes, Professor Dewar, and others as to the exceptionally good quality of the water on the occasions on which they had examined it was very much beside the mark, for the more completely the companies prove that they are able to furnish a pure water, the greater, one would think, their condemnation when "infusoria, diatoms, and other living organisms" find their way into the pipes. Now, however, we come to the judgment according to which we have no right to expect pure water, seeing that the companies have a statutory right to draw from a foul source, and can only be expected to make reasonable provision for its purification. "The highest standard of purity," we are told, "could hardly be expected from water derived from the river Thames. This was the source of supply authorised by the company's special Acts, and the Court were satisfied that all steps were being taken to supply water as pure and wholesome as it could be rendered." Thus we see that it is not pure water which we can demand, but water as pure as can be expected, considering where it comes from. Now we have heard a good deal lately about the beauty of the Thames as a source of London water and of the efficiency of filtration as a means of purifying water run from so foul a source; and on the strength of the assertions so persistently made as to the possibility of securing pure water from the Thames, the schemes from time to time set forth for obtaining water from other sources have fallen rather into abeyance. It comes, then, rather as a shock to find that, so far as the law of the matter is concerned, the standard of purity which can be enforced upon the water companies is limited by the fact that they have a statutory right to draw water from a source which is admittedly impure. Years ago Sir George Buchanan said that in estimating the purity of a water supply one must seek out whence it came, and consider to what chances of pollution it had been exposed; in other words, that we must not expect that thorns will bring forth grapes. This now appears to be the legal view, and it is as well that Londoners should recognise the position in which they stand. In ordinary circumstances no doubt the filtration is very well done. But when flood water comes down, and when "infusoria, diatoms, and other living organisms" gain access to the pipes, instead of the water companies being brought to book, we are told that, as they have a statutory right to use dirty water, we have only got what we ought to expect—which is probably just the truth.

